# Psychometric analysis and linguistic adaptation of the Persian version of Contraceptive Self-Efficacy Scale (CSES-P)

**DOI:** 10.1186/s12889-024-18147-z

**Published:** 2024-03-27

**Authors:** Nadereh Azari, Hassan Mahmoodi, Saeed Mousavi, Mojgan Mirghafourvand, Razieh Keikhaee, Abdolreza Shaghaghi

**Affiliations:** 1 Health Promotion & Education Department, Faculty of Health, Tabriz University of Medical Sciences, P.C: 5166614711, Tabriz, Iran; 2 Social Determinants of Health Research Center, Research Institute for Health Development, Kurdistan University of Medical Sciences, Sanandaj, Iran; 3 Epidemiology & Biostatistics Department, Faculty of Health, Tabriz University of Medical Sciences, P.C: 5166614711, Tabriz, Iran; 4 Social Determinants of Health Research Center, Tabriz University of Medical Sciences, Tabriz, Iran; 5 Department of Midwifery, Faculty of Nursing and Midwifery, Tabriz University of Medical Sciences, Tabriz, Iran; 6 Medical Education Research Center, Health Management and Safety Promotion Research Institute, Tabriz University of Medical Sciences, P.C: 5165665811, Tabriz, Iran

**Keywords:** Contraceptive, Unintended pregnancies, Psychometric, Self-efficacy, Validation

## Abstract

**Background:**

This study was aimed to test adaptability of the Contraceptive Self-Efficacy Scale (CSES) for use on Persian-speaking women of reproductive age.

**Method:**

A preliminary draft of the Contraceptive Self Efficacy Scale (CSES) was prepared according to the standard translation/back translation procedures and an expert panel appraised its content and face validities. The approved draft was tested on 400 randomly selected women of reproductive age (15–49 years) at the 29th Bahman Hospital of in Tabriz, the capital city of East Azerbaijan province, North West of Iran from May to August 2018. The exploratory and confirmatory factor analysis (EFA, CFA) was carried out to verify the implicit factor structure of the CSES for use on Persian-speaking women of fertile age. The Cronbach’s α and Interclass Correlation coefficients were estimated for internal consistency and accuracy assessment of the instrument.

**Results:**

This translated scale indicated good internal consistency (0.9) and reliability (0.9). A four-factor solution best fitted the study data and the estimated fit indices were in the acceptable range (chi square/ degree of freedom = 2.956, the Root Mean Square Error of Approximation = 0.070, Confirmatory Fit Index = 0.667, The Tucker-Lewis Index = 0.599).

**Conclusion:**

The CSES-P can be considered as a potentially valid and reliable tool to assess contraceptive self-efficacy among Persian-speaking women. The CSES-P is a general instrument to measure overall contraceptive self-efficacy of the Iranian reproductive age women and it would also be fascinating to work on method specific self-efficacy tools in future.

**Supplementary Information:**

The online version contains supplementary material available at 10.1186/s12889-024-18147-z.

## Background

Planned and wanted pregnancies could have positive and long-lasting impacts on the health of women in child bearing ages but unintended or unsought pregnancies might cause detrimental consequences for these women [1]. Precedence of well-planned pregnancies is far beyond the women’s health and extends to cross-generational wellness in the international communities. Therefore, access to reproductive health care which was used interchangeably in recent years with family planning services has been introduced by the World Health Organization (WHO) as a part of human rights [2].

Reproductive health counseling in the modern societies is a sine qua non element of provided health care packages in urban/rural health care centers to empower women of reproductive age in better controlling of their own reproduction, surpassing perception of the risk involved in unplanned pregnancies and to facilitate informed selection and proper utilization of contraceptive choices [3]. Perceived self-efficacy (PSE) is a key factor for empowerment of women for better reproductive health profile and those with a higher level of PSE tend to have more positive attitude toward active participation in reproductive health programs and present a higher success rate in overcoming challenges in front of their pregnancies’ planning [4]. Conversely, people with lower level of PSE are less likely to adapt favorable reproductive health behaviours or to have sufficient enthusiasm for accomplishment of reproductive health responsibilities [5].

Robust research evidence suggests crucial role of the contraceptive PSE in preventing unintentional pregnancies among reproductive-age women [6] and accusation of other related health promoting behaviours [6, 7]. It was also revealed that those with higher level of PSE are more likely to have stable behavioural pattern after adoption of new health behaviours [8].

Several studies were conducted to assess association between PSE and contraceptives’ application [9] and also success rate in prevention of unplanned pregnancies [10, 11] in different countries however, general self-efficacy scales were applied in these studies with potential probability of data collection bias. Reliable measurement of contraceptive self-efficacy is an intrinsic step forward to improve appositeness of the scientific evidence for utilization in research and practice settings in Iran and other Persian-speaking sub-groups of women living across the world.

CSES introduced by Levinson [12] for explicit application in contraceptive self-efficacy assessment and its psychometric properties were appraised for use in different populations including Mexicans [13], Chinese [14], and Americans [15, 16] with almost identical extracted factorial structure in accord with the original questionnaire. To the best of the current knowledge a contraceptive specific instrument to measure self-efficacy of the Persian-speaking women of reproductive age do not exist which could hinder reliable measurement of their preparedness in adapting behaviours that could contribute to an ameliorated quality of life. This is especially important in traditionally masculine societies where gender stereotypes are deeply entrenched in the normative believes, are justified and legitimized and contribute to widespread violation of women’s rights [17–19].

### Purpose of current study

Considering the mediating role of contraceptive self efficacy in improving reproductive health of women and their pregnancy-related informed decision making this cross-sectional psychometric study was aimed to appraise psychometric properties of the CSES-P for application in research and probably practice settings to quantify contraceptive self-efficacy of the Iranian and potentially other Persian-speaking women of reproductive age.

## Methods

### Participants

The study sample included 400 women of fertile age (15–49 years) who had an elective/non-elective delivery and admitted in the postnatal ward of the 29th Bahman Hospital in Tabriz, North West of Iran from 27 May to 1 August 2018.

### Sampling procedures

The study sample size was determined based on the Comrey and Lee’s [20] recommended sample size range for scales’ validation studies. According to their offered sample sizes for executing factor analysis a sample size of 100 is barely adequate, sample size of 200 is fairly sufficient, sample size of 300 is satisfactory, sample size of 500 is outstanding and sample size of 1,000 or more is exquisite [20]. Conforming to the available logistics and time for conducting this research, the stratified sampling method was applied to recruit 333 urban and 67 rural women (*n* = 400) based on the ratio of admitted rural/urban women in the previous Iranian fiscal year (March 2017- March 2018). Random sampling method was applied to select the study participants from the strata and for those who had not the inclusion criteria next person in the list was selected as replacement.

### Measure

Contraceptive self-efficacy of the study attendants was assessed using the Persian translated version of the CSES (CSES-P) that has 18 items which are rated by a respondent on a 5-point Likert-type scale ranging from 1 = not at all true of me, 2 = slightly true of me, 3 = somewhat true of me, 4 = mostly true of me to 5 = completely true of me. The scale’s items 1, 3, 4, 7, 10, 13, 14, 15, 16, 18 were suggested by the scale developer to be scored 1–5 and items numbered 2, 5, 6, 8, 9, 11, 12 and 17 to be reverse coded due to their negative wordings (i.e. 5 − 1) [12]. The item 14 which refers to autonomous application of vaginal diaphragm or contraceptive foam during sexual intercourse was removed from the items’ list since; they are not being provided in the country’s contraceptives market. Thus, every individual attendant was eligible to obtain an average total score values within the range of 17–85 with higher score indicative of greater perceived contraceptive self-efficacy.

### Translation procedures of the CSES

Standard translation/back translation procedures were followed to prepare a Persian version of the CSES [21]. The original questionnaire was translated into Persian by a general professional English translator and also by a topic expert familiar with English language in professional level. The preliminary drafts were checked by research team at the next step for accuracy and any mismatches. The verified Persian translated drafts were back translated into English by two other general and topic expert translators. Members of the research team were scrutinized these back translated versions and the matched up version was contrasted side-by-side against the original scale. All disagreements and mismatches were corrected at this stage and the final CSES-P was approved linguistically for further rehearsal.

### Validity of the CSES-P

Face validity of the CSES-P draft was appraised by a panel of experts consisting 16 professional informants in terms of understandability, proper vocabulary and syntax, lucidity of the wordings and cultural adaptability. The panel members were also requested to give their comments about necessity, relevancy, simplicity and clarity of items on a validity assessment form in 4-point Likert scale format to assure the scale’s content validity and consistency with its application objective. Based on the panelists’ given responses the introduced Lawshe [22] Content Validity Index (CVI) and Content Validity Ratio (CVR) were calculated to assess the panelists’ proportional level of agreement on the scale’s items. Quantitative validity of the translated CSES was assessed by conducting factor analysis. The Kaiser-Meyer-Olkin (KMO) test of sampling adequacy and Bartlett’s test of sphericity were performed and the KMO value above 0.7 and statistically significant results of Bartlett’s test were deemed as indicative of the study data factorability [23]. Principal Component Analysis (PCA) with Varimax rotation was utilized in the EFA to find simple loadings for oblique factors. The applied software for data analysis were SPSS ver-25 [24] and AMOS version 22 [25].

### Reliability of the CSES-P

Internal consistency measure of Cronbach’s α with acceptable value of above 0.7 [26] and test-retest intra-class correlation coefficient (ICC) based on the collected data from 30 eligible women at 10 days interval and in the acceptable range of equal or greater than 0.7 [27] were estimated.

### Data collection procedures

Face to face interviews were performed after explanation of the study objectives, the opted privacy, anonymity and precaution procedures to the admitted women for delivery in the past 24–48 h in the hospital’s postpartum ward. A specific time was set aside for answering the interviewees’ probable questions before obtaining their informed consent and start of the interview session especially for the respondents with low literacy level.

## Results

The mean age of the interviewed women was 28.8 years old with standard deviation of 5.7. The highest educational qualification of the interviewees was high school diploma in 65% of cases, 90% were housewives and about 42.6% reported to have acceptable level of monthly income (Table 1). Unintendedness of recent pregnancy was self-reported by about 24.7% of the respondents and almost 21.2% of them were in believe that their partners had not intention to conceive before their recent pregnancy (Table 1).

**Table 1:** Socio-demographic features of the studied Iranian women (*n* = 400, mean age = 28.8, SD = 5.7)

Variables			Number (%)
**Education**			
	Primary		23 (5.8)
	Secondary		70 (17.5)
	High School		47 (11.7)
	Diploma		172 (43.0)
	Higher degree		88 (22.0)
**Type of occupation**			
	Housewife		361 (90.2)
	Work at home		15 (3.8)
	Work outside home		24 (6.0)
**Residence place**			
	Urban		333 (83.3)
	Rural		67 (16.7)
**Income level**			
	Adequate		77 (19.3)
	Inadequate		97 (24.3)
	Fairly adequate		226 (56.4)
**Intentionality of the recent pregnancy based on the respondents’ point of views**			
	Yes		301 (75.25)
	No		99 (24.75)
**Intentionality of the recent pregnancy based on the partners’ willingness to conceive**			
	Yes		315 (78.75)
	No		85 (21.25)
**Mean number of pregnancies/children**			
		Mean number of pregnancies	2.1 (0.9)^*^
		Mean number of children	1.8 (0.6)^*^
		Mean number of sons	1 (0.7)^*^

* = Standard Deviation

The calculated CVR for the CSES-P items were in the range of 0.5–0.75 and the estimated CVR was 0.69 that represented an acceptable degree of consensus among the panelists about appropriateness of the items and their relevance for the construct being measured [28] (Table 2). Values of the internal consistency measure of Cronbach’s α (0.9) and test-retest ICC (0.9) coefficients were also in the vicinity of acceptable range.

**Table 2:** The CSES-P items’ content validity ratio and the scale’s content validity index (*n* = 400)

The CSES-P items	Content Validity Ratio (CVR)
1	0.62
2	0.71
3	0.75
4	0.50
5	0.75
6	0.50
7	0.75
8	0.62
9	0.50
10	0.75
11	0.75
12	0.75
13	0.62
14	0.75
15	0.50
16	0.62
17	0.62
**Content Validity Index (CVI)**	**0.82**

Based on the findings of the EFA a four-factor model fitted the study data similar to the items’ loadings for the corresponding component in the original CSES [12] (Table 3).

**Table 3:** Factor loadings and eigenvalues of the identified four latent variables in the CSES-P (*n* = 400)

Item Number	Questions		Factor loadings			
			1	2	3	4
2		Even if a partner can talk about sex, I can’t tell a man how I really feel about sexual things.	0.591			
5		If my partner didn’t talk about the sex that was happening between us, I couldn’t either.	0.614			
6		When I think about what having sex means, I can’t have sex so easily.	0.521			
12		It would be hard for me to go to the drugstore and ask for contraceptives without feeling embarrassed.	0.431			
16		There are times when I should talk to my partner about using contraceptives; but, I can’t seem to do it in the situation.	0.581			
17		Sometimes I end up having sex with my partner because I can’t find a way to stop it.	0.448			
1		When I am with a partner, I feel that I can always be responsible for what happens sexually with him.		0.570		
13		If my partner and I were getting really heavy into sex and moving towards intercourse and I wasn’t protected I could easily ask him if he had a protection (or tell him that didn’t)		0.716		
14		If my partner and I were getting really heavy into sex and moving towards intercourse I could tell him that I was on the pill or had an IUD (if I used them for birth control).		0.637		
4		If my partner and I are getting “turned-on” sexually and I don’t really want to have sexual intercourse (go-all-the-way, get-down), I can easily tell him “No” and mean it.			0.580	
7		If my partner and I are getting “turned-on” sexually and I don’t really want to have sexual intercourse (go-all-the-way, get-down), I can easily stop things so that we don’t have intercourse.			0.561	
15		If my partner and I were getting really heavy into sex and moving towards intercourse I could stop things before intercourse, if I couldn’t bring up a subject of protection.			0.606	
3		When I have sex, I can enjoy it as something that I really wanted to do.				0.654
8		There are times when I’d be so involved sexually or emotionally, that I could have sexual intercourse even if I weren’t protected (using a form of birth control)..				0.489
9		Sometimes I just go along with what my date wants to do sexually because I don’t think I can take the hassle of trying to say what I want.				0.549
10		If there were a man (partner) to whom I was very attracted physically and emotionally, I could feel comfortable telling him that I wanted to have sex with him				0.614
11		I couldn’t continue to use a birth control method if I thought my parents might find out.				0.574

**Fig. 1:**
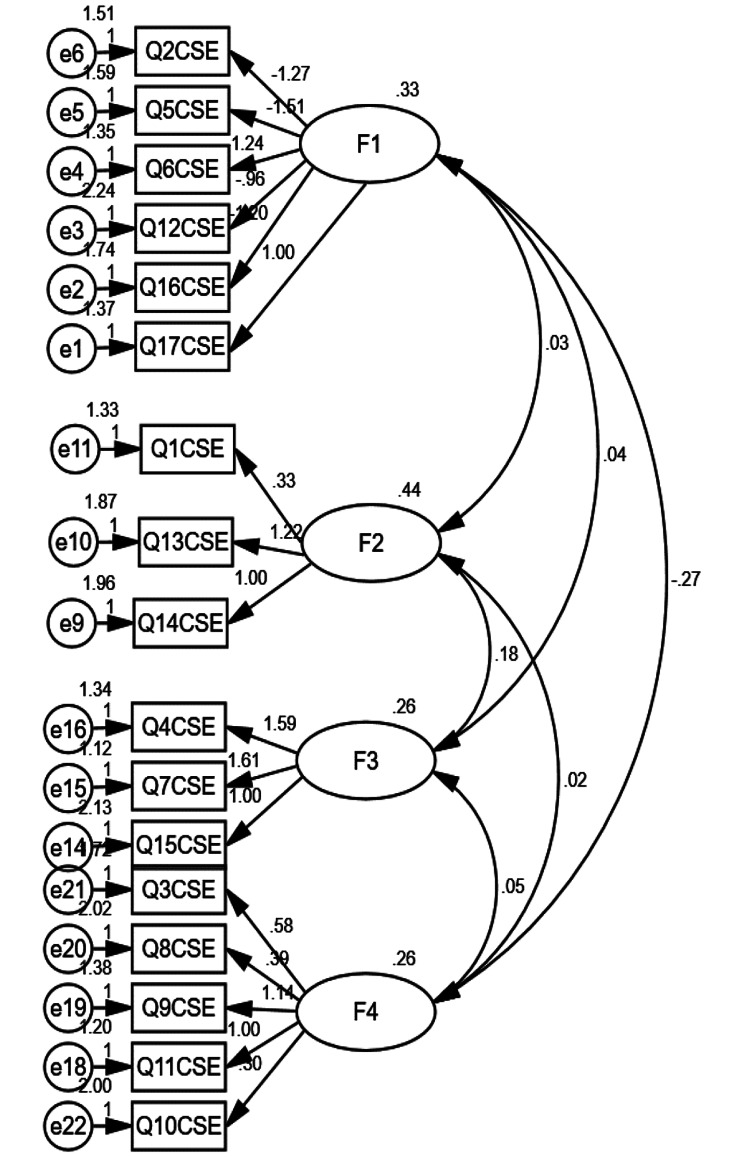
The path diagram of CFA representing factor structure of the Persian version of contraceptive self-efficacy scale (CSES-P) (*n* = 400). F 1: Planning and conscious acceptance of sexual activity. F 2: Assumption of responsibility for the direction of sexual activity and contraceptive use. F 3: Assertiveness in preventing sexual intercourse in an involved situation. F 4: Positively experiencing sexual feelings

The outputs of CFA represented desirable magnitudes (X2/df = 2.956, RMSEA = 0.07, CFI = 0.667, TLI = 0.599) for the four factor structure obtained in the EFA (Fig. 1).

The identified four latent variables were:

 (1) Planning and conscious acceptance of sexual activity (i.e., thinking and talking about sex and seeking contraception) (items 2, 5, 6, 12, 16, 17) (Cronbach’s α = 0.91).

 (2) Assumption of responsibility for the direction of sexual activity and contraceptive use (items 1, 13, 14) (Cronbach’s α = 0.88).

 (3) Assertiveness in preventing sexual intercourse in an involved situation (items 4, 7, 15) (Cronbach’s α = 0.97).

 (4) Positively experiencing sexual feelings (items 3, 8, 9, 10, 11) (Cronbach’s α = 0.97).

The estimated range of score for contraceptive self-efficacy of the studied women was 35–83 with mean score of 56.7 that represents a fairly acceptable contraceptive self-efficacy level. Employed women and those with academic qualifications obtained a higher contraceptive self-efficacy mean score (59.6, 61) than their unemployed (housewives) counterparts and those with lower educational qualifications (56.1, 56.6) (*P* <.05).

## Discussion

This study was conducted to assess psychometric properties of the Persian version of CSES for use amongst Iranian women of reproductive age. The qualitative and quantitative validity and reliability appraisal of the CSES-P approved its reliability, face and content validity for measurement of perceived contraceptive self-efficacy amongst Persian-speaking women of fertile age. The CSES-P with 17 items could be administered in a relatively short time therefore, it could be recommended for application in clinical and research settings.

The EFA and CFA outputs revealed that the identified four-factor solution corresponds well to the theoretically derived constructs in the original CSES psychometric study [12].

To the best of our current knowledge, the CSES is the only known and validated measure for contraceptive self-efficacy assessment which has been validated for use in different population subgroups [10, 12–14, 21, 29]. This study findings implied that application of the CSES-P in the Iranian socio-cultural context is feasible to assess contraceptive self-efficacy of the Persian-speaking women of childbearing age. Thus, CSES-P application could help evidence-informed interventions for reproductive health improvement of these women.

The calculated internal consistency coefficient of the CSES-P was relatively higher compared to the value obtained (0.9) in the psychometric assessment of the original CSES [12] and also two other studies on Chinese (0.65) and American women of childbearing age (0.73) [30, 31]. The estimated mean score of CSE (56.7) was almost identical to the reported value (56.4) in the study of Chinese women but lower than the obtained value (73.26) in the study of CSE on American women [29]. Inherent differences in the socio-demographic characteristics, cultural distinctions resulting to gender and power inequalities in sexual relationship of the studied women in these studies and most importantly sensitive nature of the information requested during the data collection along with conservative tents that overshadow the milieu of reproductive behaviours in philosophically different cultural compass in the Eastern and Western Hemispheres could potentially be considered as the main explanatory factors for the observed variations. Additional research is needed to determine how prior experience of women in using contraception or being talked about different contraceptive methods (formally or informally) may influence their self efficacy beliefs in distinct ways [32].

Research evidence suggested that unintended pregnancies may occur due to several factors such as lack of access to contraceptives or their inconsistent or incorrect application by couples of reproductive age [33]. However; research on the most intriguing predictors of contraceptives’ efficacy and reproductive behaviours of women and men is still topic of an open debate among scholars and policy makers [10, 26, 29]. The role contraceptive self-efficacy could play in the global sexual and reproductive health agenda is conspicuous. As a result, empowerment of women regarding their contraceptive self-efficacy rather than solely enhancing contraceptive knowledge or access must be a sine qua non ingredient of the reproductive health enhancing programs. Women’s contraceptive self-efficacy enhancement could increase their capability of maintaining more control over their reproduction and sexual behaviours and lower contraceptive failure rate in pregnancy planning [12]. The aftermath, could pose great impact on long term woman’s quality of life worldwide.

A person belief about his/her abilities in sexual decision making, sexual and contraceptive communication skills and contraceptive use [34] plays a transformative role in improving reproductive health [35]. Several strategies were introduced for enhancing CSE including boosting comprehensive contraceptive knowledge, physical and mental health development, critical consciousness improvement, sexual partners’ emotional intimacy and interpersonal communication quality enhancement, social support reinforcement, body respect encouragement, gender and reproductive norms amelioration, general and health system wide organizational culture advancement [35]. But none of these strategies outweighs the power of availability of contraceptive choices and the quality of counselling. However, empowerment of women to achieve their reproductive goals will be inconceivable without careful attention to the importance of self efficacy as a moderating variable that could cast influence on women’s reproductive success. Thence, incorporating topics such as sexual decision making and contraceptive communication skills in the routine reproductive health services could have an added bonus when paralleled with contraception freedom of choice and autonomy.

Application of a nuanced and sensitive measurement tool in studies on contraceptive self efficacy therefore, could greatly add to the value of scientific enterprises and credibility of the interventional programs.

### Limitations

This study was limited due to the probable effect that postpartum depression, irritability and mood change could potentially had upon the respondents’ answers. The socio-economic homogeneity (selection bias) of the study sample (mainly from lower socio-economic classes), self-reporting of contraceptive self-efficacy (data collection bias) and recall bias (inaccurate reporting bias) could be considered as other sources of uncertainty in this study. The possible effect of study participants’ age on their perceived contraceptive self-efficacy, their previous experiences in use of contraceptive methods and probably giving socially desirable answers rather than reporting their actual perceptions also warrants interpretation of the findings with caution. Further research is recommended to assess accuracy of the CSES-P in different ethnic socioeconomic and sub-cultural groups of Persian-speaking women in Iran, surrounding countries and worldwide.

## Conclusions

The study provided an initial exploration of knowledge about contraceptive self-efficacy measurement tool which could be applicable with the other internationally familiar instruments [33] to assess pregnancy intention of Iranian women. The findings could shed light on future direction in pre and post assessment of interventions targeting contraceptive self-efficacy promotion and devising its impact on pregnancy intentionality. Thus, health care providers would better be able to improve the efficiency and effectiveness of contraception counseling. To further explore underlying factors of contraceptive self-efficacy, qualitative studies on the contraceptive self-efficacy perceptions of the Iranian and other Persian-speaking women are recommended. The CSES is a general instrument to measure overall contraceptive self-efficacy of reproductive age women and it would also be fascinating to work on method specific self-efficacy tools in future.

### Electronic supplementary material

Below is the link to the electronic supplementary material.

Supplementary Material 1

## Data Availability

The datasets used and/or analyzed during the current study are available from the corresponding author on reasonable request.

## Abbreviations

CFI: Comparative Fit Index
CVI: Content Validity Index
CVR: Content Validity Ratio
CSES: Contraceptive Self-Efficacy Scale
EFA: Exploratory factor analysis
ICC: Intra-class correlation coefficient
KMO: Kaiser-Meyer-Olkin
PCA: Principal Component Analysis
RMSEA: Root Mean Square Error
TLI: Tucker-Lewis index
WHO: World Health Organization
