# Supplementary material for: Psychometric analysis and linguistic adaptation of the Persian version of Contraceptive Self-Efficacy Scale (CSES-P)
Source: BMC Public Health. 2024 Mar 27;24:906. doi: 10.1186/s12889-024-18147-z (PMC10967056; doi:10.1186/s12889-024-18147-z)
Supplement: Supplementary file 1 — Supplementary Material 1 [file 12889_2024_18147_MOESM1_ESM.pdf]

Contraceptive Self-Efficacy Scale

The items on the following page are a list of statements. Please rate each item on a 1 to 5 scale according to how true the statement is of you. Using the scale, circle one number for each question.

1= Not at all true of me

2= Slightly true of me

3= Somewhat true of me

4= Mostly true of me

5= Completely true of me

1. When I am with a partner I feel that I can always be responsible for what happens sexually with him/her.	1	2	3	4	5
2. Even if a partner can talk about sex, I can't tell him/her how I really feel about sexual things	1	2	3	4	5
3. When I have sex, I can enjoy it as something that I really wanted to do.	1	2	3	4	5
4. If my partner and I are getting "turned on" sexually and I don't really want to have sexual intercourse, I can easily tell her/him "No" and mean it.	1	2	3	4	5
5. If my partner didn't talk about the sex that was happening between us, I couldn't either.	1	2	3	4	5
6. When I think about what having sex means, I can't have sex so easily.	1	2	3	4	5
7. If my partner and I are getting "turned on" sexually and I don't really want to have sexual intercourse (go all the way, get down), I can easily stop things so that we don't have intercourse.	1	2	3	4	5
8. There are times when I'd be so involved sexually or emotionally that I could easily have sexual intercourse even if I weren't protected (using a form of birth control).	1	2	3	4	5

This instrument is copyrighted by the author and is reproduced here with permission.
Permission from the author is required prior to use of this tool.
Please address correspondence to Ruth Andrea Levinson: rlevinso@skidmore.edu.

Contraceptive Self-Efficacy Scale (continued)

1= Not at all true of me

2= Slightly true of me

3= Somewhat true of me

4= Mostly true of me

5= Completely true of me

9.	Sometimes I just go along with what my date wants to do sexually because I don't think I can take the hassle of trying to say what I want.	1	2	3	4	5
10.	If there were a man (boyfriend) to whom I was very attracted physically and emotionally, I could feel comfortable telling him that I wanted to have sex with him.	1	2	3	4	5
11.	I couldn't continue to use a birth control method if I thought that my parents might find it.	1	2	3	4	5
12.	It would be hard for me to go to the drugstore and ask for foam (Encare Ovals, a diaphragm, a pill prescription, etc.) without feeling embarrassed.	1	2	3	4	5
13.	If my boyfriend and I were getting really heavy into sex and moving towards intercourse and I wasn't protected . . .					
a.	I could easily ask him if he had protection (or tell him that I didn't).	1	2	3	4	5
b.	I could excuse myself to put in a diaphragm or foam (if I used them for birth control).	1	2	3	4	5
c.	I could tell him I was on the pill or had an IUD (if I used them for birth control).	1	2	3	4	5
d.	I could stop things before intercourse, if I couldn't bring up the subject of protection.	1	2	3	4	5
14.	There are times when I should talk to my boyfriend about using contraceptives, but I can't seem to do it in the situation.	1	2	3	4	5
15.	Sometimes I end up having sex with a boyfriend because I can't find a way to stop it.	1	2	3	4	5

This instrument is copyrighted by the author and is reproduced here with permission.
Permission from the author is required prior to use of this tool.
Please address correspondence to Ruth Andrea Levinson: rlevinso@skidmore.edu.
